# Caregiver Burden in Distance Caregivers of Patients with Cancer

**DOI:** 10.3390/curroncol29110704

**Published:** 2022-11-21

**Authors:** Sumin Park, Susan R. Mazanec, Christopher J. Burant, David Bajor, Sara L. Douglas

**Affiliations:** 1 Frances Payne Bolton School of Nursing, Case Western Reserve University, Cleveland, OH 44106, USA; 2 Case Comprehensive Cancer Center, Case Western Reserve University, Cleveland, OH 44106, USA; 3 University Hospitals Seidman Cancer Center, Cleveland, OH 44106, USA; 4 Geriatric Research Education and Clinic Center, Louis Stokes VA Medical Center, Cleveland, OH 44106, USA; 5 School of Medicine, Case Western Reserve University, Cleveland, OH 44106, USA

**Keywords:** distance caregiver, cancer, burden, psychological symptom

## Abstract

Distance caregivers (DCGs), those who live more than an hour away from the care recipient, often play a significant role in patients’ care. While much is known about the experience and outcomes of local family caregivers of cancer patients, little is known about the experience and outcomes of distance caregiving upon DCGs. The purpose of this study was to identify the relationships among stressors (patient cancer stage, anxiety, and depression), mediators (DCG emotional support and self-efficacy), and burden in DCGs’ of patients with cancer. This study was a descriptive cross-sectional study and involved a secondary data analysis from a randomized clinical trial. The study sample consisted of 314 cancer patient–DCG dyads. The results of this study were: (1) 26.1% of DCGs reported elevated levels of burden; (2) significant negative relationships were found between mediators (DCG emotional support and self-efficacy) and DCG burden; and (3) significant positive relationships were found between patient anxiety, depression, and DCG burden. The prevalence of burden in DCGs, and its related factors, were similar to those of local caregivers of cancer patients, which suggests that interventions to reduce burden in local caregivers could be effective for DCGs as well.

## 1. Introduction

In the United States, approximately seven million family caregivers are managing patients’ care from a distance, and it is anticipated to continue to increase over time [1]. Providing patient care from a distance adds additional complexity and stress to the caregiving role [1]. Distance caregivers (DCGs)–those living more than an hour away from the care recipient–experience negative emotions (a sense of guilt, feelings of doubt or being incapable of being a successful caregiving) and financial strain due to interrupted work and travel expenses, which ultimately can lead to an increase in caregiver burden [1,2,3]. Geographical distance, a lack of information regarding the patient’s condition during their illness trajectory, and a deficiency of support from healthcare providers increase the psychological burden in DCGs as well [2]. Furthermore, in a national study, 23% of DCGs were the sole or primary caregiver, 65% visited the patient at least once a month, and 46% spent time scheduling services for the patient [3]. These findings demonstrate the involvement of DCGs in patients’ physical and emotional care even though they may be physically distanced from the patients.

**Figure 1:**
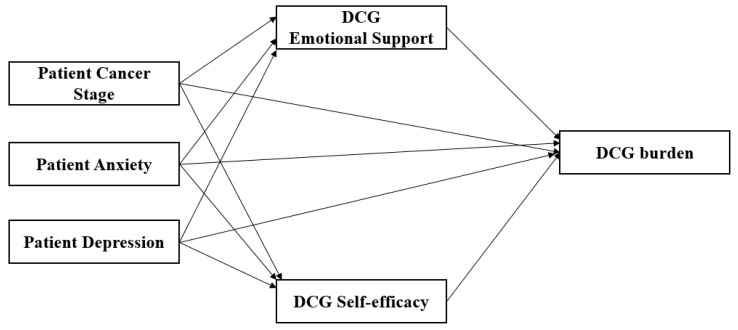
Study conceptual framework.

It has been established that local caregivers of cancer patients experience a high level of caregiver burden, which, in turn, relates to poor physiological and psychological outcomes [4,5]. A patient’s functional status and psychological symptoms, including anxiety and depression, are known to be related to caregiver burden [4,5]. In addition, low emotional support and self-efficacy in family caregivers are contributing factors to high caregiver burden [6]. To date, the majority of research has focused on family caregivers of cancer patients who live near the patient and are providing day-to-day care or seeing the patient on a regular basis (known as local caregivers). However, research has shown that DCGs report extreme psychological burden that is as high or even higher than their local counter parts [2]. Therefore, it is important to assess DCGs’ psychological well-being (such as caregiver burden) and to identify factors that contribute to high caregiver burden. Research that focuses on the DCGs of patients with cancer is sparse, yet a qualitative study has identified key contributors to DCG stress, which are uncertainty, a lack of communication, a lack of emotional support and a lack of self-confidence in their ability to be an effective caregiver [2]. Therefore, it is important to examine the relationships between and among factors that can potentially minimize these sources of stress for DCGs in the hope of reducing negative psychological outcomes. The aim of this study was to identify the relationships among stressors (patient cancer stage, anxiety, and depression), mediators (DCG emotional support and self-efficacy), and burden in distance caregivers of patients with cancer. The conceptual framework of the study is presented in Figure 1. This study was guided by the Stress-Appraisal Model of Caregiving, which defined caregiver burden as an appraisal of the situation that is affected directly and indirectly by stressors (physical and/or emotional condition of care recipients) and resources [6].

## 2. Materials and Methods

The study was a descriptive cross-sectional study, conducting secondary data analysis from a parent study “CLOSER_A Videoconference Intervention for Distance Caregivers (DCGs)”. The parent study was a three-group randomized clinical trial that was examining the effectiveness of interventions using videoconference technology involving patients with cancer and their DCGs (NCT02666183). The parent study recruited subjects from May 2016 to October 2019 at the outpatient clinics at the Seidman Comprehensive Cancer Center at University Hospitals Cleveland Medical Center in Cleveland, Ohio. Baseline data were used for the present study analyses.

### 2.1. Sample

The sampling frame was comprised of DCGs (family members who lived >one hour travel time away from the patient) of patients with cancer. A convenience sampling method was used for the parent study recruitment of subjects. Patient eligibility criteria were: (1) adult patient (>18 years) diagnosed with solid tumor cancer; (2) actively receiving treatment; (3) able to speak and understand English; (4) has more than six-month life expectancy; and (5) has a DCG involved in patient care, support, and/or care planning. DCG eligibility criteria were: (1) adult caregiver (>18 years) of cancer patient; (2) lives ≥ an hour travel time away from patient; (3) able to speak and understand English; and (4) has access to Internet (smartphone, computer, etc.). Power analysis for this study was conducted based upon the primary aim of the study—to examine whether emotional support and self-efficacy mediated the relationship between stressors (patient cancer stage, anxiety, and depression) and DCG burden. Using an alpha of 0.05, power of 0.90, effect size of 0.15, non-directional hypothesis, and number of predictors of 6, a sample size of 123 was deemed to be sufficient.

### 2.2. Procedures

In the parent study, patients who met eligibility criteria and were interested in participating in the study were asked if they had a family member who lived more than an hour travel time away from the patient (potential DCG participant). Both patient and DCG were required to provide written consent to participate in the study. Patient data were collected by research assistants in the outpatient clinic during the patients’ oncologist visits and DCG data were sent to their emails and collected remotely using the Research Electronic Data Capture (REDCap) [7,8]. Both patient and DCG data were collected at baseline, 4 months (immediately following the intervention period), and 6 months after enrollment. Only the baseline data were used for the current study. The study had institutional approval from the University Hospital Institutional Review Board (08-15-07C). All subjects were assigned code numbers and identified solely by code numbers. Collected consents and study materials were stored in a locked office.

### 2.3. Measures 

Patients completed the following self-report questionnaires: anxiety, depression, and demographic information (age, gender, race, cancer type, education level, treatment type). Patient’s cancer stage was abstracted from the electronic medical record. DCGs completed the following self-report questionnaires: emotional support, self-efficacy, caregiver burden, and demographic information (age, gender, race, education level, socioeconomic status, employment, relationship to patient, presence of local caregiver).

#### 2.3.1. Anxiety

The PROMIS^®^ Short Form v1.0-Anxiety 4a scale was used to measure patient anxiety [9]. The scale consists of 4 items that use a 5-point Likert scale (1 = never to 5 = always). Possible total raw scores range from 4 to 20 and t-scores were computed from the raw scores, using a conversion table from the PROMIS^®^ scoring manual [9]. The total t-scores range from 40.3 to 81.6, with the mean score being 50 and standard deviation of 10. Higher scores reflect greater anxiety. The cut points are: within normal limits (<55), mild (55–59), moderate (60–69), and severe symptoms (≥70). The PROMIS^®^ anxiety scale has excellent psychometric properties [10]. Cronbach’s alpha for the scale at baseline in this study was 0.80.

#### 2.3.2. Depression

The PROMIS^®^ Short Form v1.0-Depression 4a scale was used to measure patient depression [9]. The scale, scoring and cut points for the Depression scale are the same as for the PROMIS anxiety scale and total t-scores range from 41.0 to 79.4. This measure also has excellent psychometric properties [10] and Cronbach’s alpha for the scale in this study at baseline was 0.86.

#### 2.3.3. Emotional Support

The PROMIS^®^ Short Form v2.0-Emotional Support 4a scale was used to measure DCG emotional support [9]. The scale consists of 4 items on a 5-point Likert scale (1 = never to 5 = always). Total scores (range from 4 to 20) were transformed to t-scores (range from 25.7 to 62.0). Higher scores reflect greater emotional support. The scale has good psychometric properties [11]. Cronbach’s alpha for the scale in this study at baseline was 0.92.

#### 2.3.4. Self-Efficacy

The New General Self-efficacy scale was used to measure DCG self-efficacy [12]. The scale consists of 8 items using a 5-point Likert scale (1 = strongly disagree to 5 = strongly agree). Total scores range from 8 to 40, with higher scores reflecting higher self-efficacy in achieving goals. Cronbach’s alpha for the scale in this study at baseline was 0.90. The scale has been reported to demonstrate convergent and discriminant evidence of validity [13].

#### 2.3.5. Caregiver Burden

Caregiver burden was measured using the Zarit Burden Interview (ZBI) screening scale, a psychometrically sound tool used in caregiving research [14]. The ZBI consists of 4 items that use a 5-point Likert scale (0 = never to 4 = nearly always). Total scores range from 0 to 16, with higher scores reflecting higher burden related to caregiving. Cutoff score of ≥7 represent elevated burden [15]. Correlation between the ZBI screening scale and the ZBI-22 (longer version) ranged from 0.83 to 0.93 [14]. The ZBI-22 has been reported to have excellent psychometric properties, including convergent validity [16]. Cronbach’s alpha for the scale in this study at baseline was 0.80.

### 2.4. Statistical Analysis

IBM^®^ Statistical Package for the Social Sciences (SPSS) was used to analyze the data. Descriptive statistics were used to describe patient and DCG demographic characteristics, patient clinical characteristics, and study variables. Categorical (nominal, ordinal) variables were reported as counts and percentages, and continuous (interval, ratio) variables were reported as mean, median, standard deviation, and range. Point-Biserial correlations were used to examine the relationships between the dichotomous variables (e.g., patient cancer stage) and continuous variables (e.g., DCG burden). Relationships among continuous variables were examined using the Pearson’s correlation. Path analysis was planned to test the mediating effect of DCG emotional support and self-efficacy between stressors (patient cancer stage, anxiety, and depression) and DCG burden. Assumptions for statistical tests were met.

## 3. Results

The study sample consisted of 314 cancer patient–DCG dyads. Demographic characteristics of the DCGs and patients are presented in Table 1. DCGs were, on average, middle aged, female, white, employed, and the adult children of the patient. Almost half had a household income of USD 100,000 or greater with a majority having a college education or greater. Patients were, on average, in their mid-60’s, female, and white. The majority of patients had metastatic stage cancer, with gastrointestinal and gynecological/genitourinary cancer being the most common types of cancer in the sample. 

As seen in Table 2, average scores for patient anxiety and depression were towards the low range of possible *t*-scores. More than a quarter of patients had anxiety symptoms out of normal range and less than 20% of patients had depressive symptoms out of normal range. Scores for emotional support and self-efficacy were on the middle to the high range of possible scores. Scores for caregiver burden were toward the low range of possible scores. In addition, more than a quarter of the DCGs felt some degree of uncertainty about what to do about the patient.

There were significant negative relationships between DCG burden and two mediators, DCG emotional support (*r* = −0.32, *p* < 0.001, 95% CI (−0.42, −0.22)) and DCG self-efficacy (*r* = −0.23, *p* < 0.001, 95% CI (−0.33, −0.12)). Patient anxiety (*r* = 0.15, *p* = 0.004, 95% CI (0.04, 0.26)) and depression (*r* = 0.21, *p* < 0.001, 95% CI (0.10, 0.32)) had significant positive relationships with DCG burden. There were no significant relationships between stressors of being a DCG (patient cancer stage, anxiety, and depression) and the two mediators (DCG emotional support and self-efficacy). There was no significant relationship between patient cancer stage and DCG burden. Mediation effect was not tested because the assumption for mediation analysis (significant relationship between stressors and mediators) was not met.

## 4. Discussion

The first key finding is that 26.1% of DCGs reported elevated burden scores—a finding similar to those reported in studies of local caregivers of cancer patients [5,15,17,18]. In addition, our study supports work of others [2] that identified DCG uncertainty as the most important factor contributing to their burden score. Therefore, healthcare providers should assess whether there are DCGs involved in the patient’s care and provide similar support to DCGs as to local caregivers. Such activities (e.g., communicating with the DCG about the patient’s status and treatment plan) can serve to also reduce uncertainty—a key contributing factor to DCG burden.

The second key finding from this study is the negative and small-moderate relationships found between two mediators (DCG emotional support and self-efficacy) and DCG burden. These findings are consistent with findings from prior studies involving local caregivers of cancer patients [18,19,20]. As a result of findings with local cancer caregivers, interventional studies aimed at reducing the burden of caregivers of local cancer patients have been developed and have focused upon psychoeducational strategies (primarily focused on improving caregivers’ self-efficacy) or supportive care strategies (focused on providing emotional support to caregivers) [21,22,23]. Given the current study’s findings, the implementation and testing of psychoeducational and supportive care interventions for DCGs of patients with cancer should be undertaken to identify existing interventions that could benefit DCGs.

The third key research finding is the significant positive and weak relationships between patient anxiety and depression and DCG burden. Although the significant direct effects found in the current study are consistent with prior research involving local caregivers of cancer patients, the strengths of the relationships are lower in the present study than what has been reported in prior research with local caregivers [4,17,20,24,25]. One potential reason for the small effect size noted in the current study is the possible existence of a moderator. Positive aspects of caregiving (e.g., sense of caregiving satisfaction) have been found to serve as moderators between patient’s psychological symptoms and caregiver burden in caregiving literature [26,27], however, this variable was not included in the current study.

There are several limitations to this study. First, the parent study used a convenience sampling method, which may limit the generalizability of study findings. Second, a cross-sectional design was used for this study which limits our understanding of DCG burden. Considering the longitudinal nature of trajectory of cancer care, factors such as the goals of care, patient symptoms, and/or treatment plans, change over time [28]. As a result, the levels of caregiver burden may vary depending on what phase of treatment and illness they are experiencing [29]. Third, variables found to relate to caregiver burden in prior studies (e.g., patient quality of life, patient performance status) [17,30] were not included in the parent study and were not available for inclusion in the current study. In order to more fully describe factors related to DCG burden, the inclusion of those variables should be considered in future research. Finally, the sample was predominantly white with high income levels, which could possibly limit the generalizability of study findings; therefore, more research should be done to include a more heterogeneous sample.

In conclusion, this study is one of the first to describe DCG burden for patients undergoing treatment for cancer and to identify factors related to caregiver burden. Like their local caregiver counterparts, DCGs experience elevated burden that is related to patient anxiety and depression and DCG emotional support and self-efficacy for caregiving. Since the prevalence of burden in DCGs and its related factors were similar to that of local caregivers of cancer patients, we suggest implementing a similar approach for DCGs which has been used to lower burden in local caregivers for DCGs (e.g., psychoeducational interventions, supportive care intervention). This work underscores the need for healthcare providers to identify DCGs and to provide information and support to this vulnerable and largely unrecognized member of the patient care team.

## Figures and Tables

**Table 1:** Patient and DCGs demographic characteristics and clinical data (*N =* 314).

Characteristics	Patients	DCGs
	*M* (*SD*)	*M* (*SD*)
Age (years)	65.31 (12.55) (Range: 25–89)	46.69 (12.54) (Range: 20–79)
	*n* (%)	*n* (%)
Gender: Female	201 (64.0)	220 (70.1)
Race: White	207 (66.3)	207 (66.1)
Ethnicity: Non-Hispanic	272 (86.6)	253 (83.8)
Education: College or greater	172 (55.5)	244 (78.2)
Marital Status: Married/Living with partner	166 (53.1)	208 (66.8)
Type of Cancer		
Gastrointestinal	115 (36.6)	
Gynecological/Genitourinary	73 (23.3)	
Breast	66 (21.0)	
Lung/Thoracic	41 (13.1)	
Other (Head and Neck, Skin, Sarcoma)	29 (6.0)	
Stage of Disease: Metastatic	250 (82.8)	
Annual Household Income		
USD 49,999 or less		73 (24.3)
USD 50,000 to USD 99,999		87 (29.0)
USD 100,000 or greater		140 (46.7)
Employment: Employed		259 (82.5)
Relationship to Patient: Child		200 (63.7)
Presence of Local Caregiver: Yes		248 (79.0)
Previous DCG Experience: No		272 (87.2)

**Table 2:** Descriptive summary of study variables (*N =* 314).

Variable	*M* (*SD*)	*Mdn*	Range	95% CI
Patient Anxiety	48.61 (8.46)	48.00	40.3–73.3	(47.67, 49.55)
Patient Depression	47.32 (7.83)	47.00	41.0–79.4	(46.45, 48.19)
DCG Emotional Support	55.09 (7.39)	55.60	34.0–62.0	(54.27, 55.91)
DCG Self-efficacy	33.25 (4.11)	32.00	21–40	(32.79, 33.39)
DCG Burden	4.49 (3.23)	4.00	0–15	(4.13, 4.85)

Note. CI = confidence interval; potential range for each measure: patient anxiety (40.3–81.6), patient depression (41.0–79.4), DCG emotional support (25.7–62.0), DCG self-efficacy (8–64), DCG burden (0–16).

## Data Availability

The de-identified data represented in this study are available on specific-request from one of the co-authors: Sara L. Douglas.
